# Nitrous Oxide Emissions from Paddies: Understanding the Role of Rice Plants

**DOI:** 10.3390/plants9020180

**Published:** 2020-02-02

**Authors:** Arbindra Timilsina, Fiston Bizimana, Bikram Pandey, Ram Kailash Prasad Yadav, Wenxu Dong, Chunsheng Hu

**Affiliations:** 1 Key Laboratory of Agricultural Water Resources, Hebei Key Laboratory of Soil Ecology, Center for Agricultural Resources Research, Institute of Genetics and Developmental Biology, Chinese Academy of Sciences, Shijiazhuang 050021, China; bizifis@gmail.com (F.B.); dongwx@sjziam.ac.cn (W.D.); 2 University of Chinese Academy of Sciences, Beijing 100049, China; bikram@cib.ac.cn; 3 Key Laboratory of Mountain Ecological Restoration and Bio-resource Utilization and Ecological Restoration Biodiversity Conservation Key Laboratory of Sichuan Province, Chengdu Institute of Biology, Chinese Academy of Sciences, Chengdu 610041, Sichuan, China; 4 Central Department of Botany, Tribhuvan University, Kirtipur 44613, Kathmandu, Nepal; rkp.yadav@cdbtu.edu.np

**Keywords:** anoxia, hypoxia, mitochondria, nitric oxide, nitrous oxide, paddy, potential pathway, rice plant

## Abstract

Paddies are a potential source of anthropogenic nitrous oxide (N_2_O) emission. In paddies, both the soil and the rice plants emit N_2_O into the atmosphere. The rice plant in the paddy is considered to act as a channel between the soil and the atmosphere for N_2_O emission. However, recent studies suggest that plants can also produce N_2_O, while the mechanism of N_2_O formation in plants is unknown. Consequently, the rice plant is only regarded as a channel for N_2_O produced by soil microorganisms. The emission of N_2_O by aseptically grown plants and the distinct dual isotopocule fingerprint of plant-emitted N_2_O, as reported by various studies, support the production of N_2_O in plants. Herein, we propose a potential pathway of N_2_O formation in the rice plant. In rice plants, N_2_O might be formed in the mitochondria via the nitrate–nitrite–nitric oxide (NO_3_–NO_2_–NO) pathway when the cells experience hypoxic or anoxic stress. The pathway is catalyzed by various enzymes, which have been described. So, N_2_O emitted from paddies might have two origins, namely soil microorganisms and rice plants. So, regarding rice plants only as a medium to transport the microorganism-produced N_2_O might be misleading in understanding the role of rice plants in the paddy. As rice cultivation is a major agricultural activity worldwide, not understanding the pathway of N_2_O formation in rice plants would create more uncertainties in the N_2_O budget.

## 1. Introduction

Nitrous oxide (N_2_O) is a major anthropogenic greenhouse gas and the single most important contributor to stratospheric ozone depletion [1,2]. It accounts for approximately 6% of the enhanced global warming effect [3]. Among the anthropogenic sources of N_2_O, the agriculture sector represents the largest source [4]. Rice (*Oryza sativa*) farming plays an important role in the agricultural sector as it is a staple food for one-half of the world’s population [5]. Additionally, rice farming occupies about 158.5 million hectares of the world’s arable land and is one of the most important economic activities that provides a livelihood to millions of people [5]. Paddies are also a contributor of N_2_O to the atmosphere [5,6]. Moreover, the paddy utilizes one-seventh of the nitrogen (N) fertilizer and one-third of irrigation globally [7], making a more potent zone of N_2_O formation, as the N fertilizer application [8] and irrigation management practices [9] contribute significantly to N_2_O emissions. So, global N_2_O emissions from the paddy might increase considerably [10]. Thus, it is necessary to understand the mechanisms of N_2_O production in the paddy for necessary steps to be taken towards mitigation strategies for the global warming effect.

N_2_O in the paddy is emitted by the soil [10,11] and the rice plant [12,13]. The processes involved in the soil are well studied [10,11]. However, processes involving the plant are overlooked and the N_2_O emitted by the rice plants in paddies is considered to be produced by soil microorganisms, with the rice plant hypothesized to act as a channel to emit it into the atmosphere [12,13]. However, studies hypothesizing rice plants as a channel to emit N_2_O [12,13] have only measured the flux from the soil and plants and concluded that rice plants are not a source of N_2_O. Recently, Lenhart et al., (2019) [14] reported the distinct dual isotopocule fingerprint of N_2_O (δ^15^N, δ^15^N^sp^ and δ^18^O) emitted from the plant *Miscanthus sinensis*, and suggested that plants are a natural source of N_2_O. So, in our opinion, measuring only the N_2_O fluxes from the soil and the rice plant may not be sufficient to prove that rice plants are not a source of N_2_O. The results of Smart and Bloom (2000) [15] do not support the hypothesis that N_2_O emitted by wheat plants is produced by microorganisms and via the transpiration process. To elucidate this, we suggest the use of the ^15^N natural abundance method by injecting ^15^N-labelled N_2_O into the soil zone and, subsequently, measuring the fluxes and the ^15^N natural abundance of N_2_O from the soil and plants to reveal whether the N_2_O emissions are emitted either from soil and plants transport them, or whether N_2_O emissions can also be produced from plant cells. Furthermore, the rice plants should be aseptically grown in a controlled hydroponic solution with a regulated O_2_ partial pressure, and NO_3_ and NH_4_ concentrations and, subsequently, the N_2_O fluxes should be measured. Moreover, recent studies have shown that various species of plants can produce N_2_O and emit it into the atmosphere [14,15,16], however, the mechanism of N_2_O formation is not clearly understood. Therefore, considering rice plants only as a channel for soil-produced N_2_O might mislead understanding of the role of rice plants in N_2_O emission, as it is a conclusion based on studies that just measured the N_2_O fluxes from rice soil and the rice plant.

There are reports of both higher [17,18] and lower flux rates of N_2_O from paddies [19,20]. The high or low emissions of N_2_O from paddies depend on the management practices [18,19,20]. The N fertilizer application rate and water level are major factors controlling the N_2_O emissions from paddies [8,20]. For example, a meta-analysis by Zou et al. (2007) [19] revealed that under a continuous flooding system, N_2_O is emitted only after drainage, whereas flooding-midseason drainage-reflooding management triggers a substantial N_2_O emission. Moreover, flooding-midseason drainage-reflooding moist intermittent irrigation practices without waterlogging trigger a threefold higher N_2_O emission than the flooding-midseason drainage-reflooding management practice. A recent meta-analysis [9] reported a 105% increase in N_2_O emission with non-continuous flooding management rather than continuous flooding. Similarly, midseason drainage and N application significantly increased the N_2_O emissions from the paddy [11]. Another meta-analysis, based on the comparison between flooding irrigation and non-flooding irrigation (reduced irrigation with midseason drainage) and N fertilizer input, showed an increase in N_2_O emission of 84.4% and 167.3%, respectively [8]. From these observations of the meta-analyses, it can be concluded that water level management and N application significantly affect the N_2_O fluxes from the paddies. Interestingly, water level management [13] and nitrogen fertilizer application [12] also significantly increased the N_2_O emission from the rice plants and, in both cases, the emissions of N_2_O were higher from rice plants than from the soil–water surface [12,13]. These results highlight the role of rice plants in N_2_O emissions from paddies.

To mitigate the effects of global warming and ozone depletion effectively, a good understanding of all of the sources of N_2_O and the regulating factors is crucial. There have been numerous studies on paddies regarding their N_2_O emissions [8,12,13,20,21], however, no study has highlighted the role of the rice plant as a source of N_2_O. Thus, it is essential to explore the role of the rice plant in paddies—that is, whether it acts as a source of or a medium to channel N_2_O. The production of N_2_O in ascetically grown plants [16,22] suggests N_2_O can be produced by even eukaryotic plant cells. Moreover, based on the distinct dual isotopocule fingerprint of plant-emitted N_2_O, Lenhart et al. (2019) [14] proposed that plants are a natural source of N_2_O. So, the rice plant might also be a source of N_2_O. Now arises the question of what the mechanism of N_2_O formation in the rice plant cell is, if the rice plant is a source of N_2_O. Due to the unknown mechanisms of N_2_O formation in the plant cell, the rice plant might be regarded as just a medium to transport the soil microorganism-produced N_2_O. Therefore, to elucidate the origin of N_2_O emitted from paddies, we propose a potential pathway of N_2_O formation within the rice plant.

## 2. Potential Pathway of N_2_O Formation in Rice Plants

Studies on plants’ N_2_O emissions, involving the usage of the ^15^N isotope labeling method, have shown nitrate (NO_3_) as a precursor to N_2_O formation, but not ammonium (NH_4_) [8,14,15,16,22,23,24]. Furthermore, aseptically grown plants and axenic algal cells, when supplied with ^15^N-NO_2_, have also been found to produce δ ^15^N-N_2_O^bulk^ [16,22,25]. In addition, eukaryotic cells, when supplied with ^15^N-NO, produce δ ^15^N-N_2_O^bulk^ via a reduced form of cytochrome c oxidase [26]. Therefore, NO_3_, NO_2_, and nitrous oxide (NO) are the sources of N_2_O in eukaryotic cells. Thus, N_2_O formation in the cells of rice plants might occur via the NO_3_-NO_2_-NO pathway.

Nitric oxide (NO), being a signaling molecule, is formed at the cellular level in every eukaryotic organism [27]. There are, mainly, two potential pathways of NO formation in cells, namely the oxidative and reductive pathways. The oxidative pathway is L-arginine-dependent and occurs when the oxygen concentration in the cells is sufficient, whereas the reductive pathway is NO_3_ and NO_2_-dependent and occurs at a low oxygen concentration in the cells [28,29]. Briefly, the reductive pathway can occur in the hypoxic or anoxic cell environment, via NO_3_-NO_2_, and is catalyzed by various enzymes [27,28,30]. For example, NO_3_ taken by the plant root is reduced to NO_2_ in the cytoplasm by the cytoplasmic nitrate reductase (NR) [29]. Hypoxia caused by flooding in the root may increase [31], decrease [32], or have no effect [33] on NO_3_ uptake from the soil. However, flood-tolerant species such as rice might have higher NR activities than flood-sensitive species [34]. During hypoxia and anoxia, the NO_2_ and NH_4_ assimilation to amino acids strongly decreases [35]. So, NO_2_ formed in the cytosol, due to the reduction in NO_3_ by NR, is transported to the mitochondria by a protein similar to that in the chloroplast [30,36]. Moreover, the mitochondrial inner membrane anion channel may also be responsible for the transport of nitrite to the mitochondria [30].

It is evident that the mitochondrion is a site of NO_2_ reduction [30,37]. NO_2_ in the mitochondria is reduced to NO, and the process is pronounced under hypoxic to anoxic stress [30,38,39] and is more favorable at a lower pH [40,41]. Accordingly, Klepper (1987) [42] and Rockel et al. (2002) [43] reported a high emission of NO from plant leaves after the supply of NO_3_ under anaerobic conditions. The reduction process in the mitochondria is catalyzed by various electron transport chains (ECTs) [39,44]. For example, complex III [45] (Benamar et al., 2008), cytochrome c [44,46], and alternative oxidase (AOX) [47] can catalyze the reduction of NO_2_ to NO in the mitochondria. The NO subsequently formed is further reduced to N_2_O by a reduced form of eukaryotic cytochrome c oxidase [26,48]. The conversion of NO into N_2_O by a reduced form of cytochrome c oxidase is more favorable at low levels of both NO and O_2_ [48]. Cytochrome c oxidase in aerobic organisms is considered to be evolved from bacterial denitrifying enzymes [49]. Therefore, it is believed that, under the condition of less oxygen in cells, the enzyme exhibits some rudimentary nitrite and nitric oxide reductase activities [26,48,50]. Moreover, quinone in the mitochondria can also catalyze the reduction of NO to N_2_O [51,52,53].

**Figure 1:**
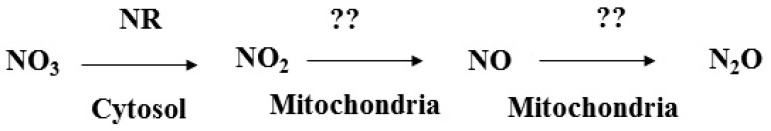
The potential pathway of N_2_O formation in rice plants under hypoxic conditions. ^??^ sign represents the involvement of two or more enzymes [9,23,27,44,48,52].

Therefore, the processes, like the reduction of NO_2_ to NO and NO to N_2_O, are favorable under hypoxic and anoxic conditions in the mitochondria of plants. As discussed in the introduction section, the emission of N_2_O from paddies is very high under non-continuous flooding, midseason drainage, and reduced irrigation with midseason drainage management practices. All of these practices alter the oxygen (O_2_) level significantly in the root cells of the rice plant [54,55], which may create a favorable zone for N_2_O formation. Laboratory-based measurements also showed approximately 82–92% of N_2_O emissions from rice plants under soil flooded conditions, whereas 7–24% were found under unsaturated soil water conditions [13]. Along with O_2_, NO_3_ also plays an important role in N_2_O emission from plants, as NO_3_ is the precursor to N_2_O production in plants [14−16]. The role of O_2_ in the proposed pathway is supported by high NO_2_ and NO emissions from the soybean plant [56] and N_2_O [13] from the rice plant under anaerobic conditions. Furthermore, the role of NO_3_ in the proposed pathway is supported by the emission of ^15^N-labelled NO and δ ^15^N-N_2_O^bulk^ from plants when supplied with ^15^N-NO_3_ [23]. Therefore, NO_3_^−^ concentration in the root zone and cells might affect the N_2_O emission from rice plants, as NO_3_^−^ uptake in the plant increases when its concentration in the soil solution is increased [57,58]. It might be the reason that soil fertilization with KNO_3_ greatly enhances N_2_O emission from rice plants [12]. Thus, N_2_O emitted by rice plants might be formed in hypoxic or anoxic mitochondria, as shown in Figure 1. It further suggests the existence of an incomplete denitrification (NO_3_-NO_2_-NO-N_2_O) pathway in the plant cells [16] when the oxygen level in the cells declines. This is further supported by the production of N_2_O in plants after the supply of NO_3_ and NO_2_, but not NH_4_ [14,15,16,22,23,24]. As there are many reports that the rice plant emits a substantial amount of N_2_O [12,13], and that within the plant cell there exists evidence of the NO_3_-NO_2_-NO-N_2_O pathway [26,29,30,37,48], this suggests that rice plants are a source of N_2_O. So, considering rice plants only as a channel for soil-produced N_2_O might create a gap in understanding N_2_O emission from paddies.

## 3. Why It Is a Challenge to Understand the Role of Rice Plants with Current Approaches to Methods from Field Studies?

Current methodologies involved in field-based measurements of N_2_O emissions from paddies include closed static chamber-based methods [18,59] and the micrometerological approach (the eddy covariance method) [10,60,61]. These methods include both soil and rice plants in the same system [10,18,59,60,61], making it difficult to evaluate the role of rice plants. However, for other plants with bigger stems (like trees), recent studies have developed separate chambers that help to capture the fluxes of N_2_O by avoiding soil N_2_O emissions [62]. The chamber used for trees cannot be used for crop species, like rice. The studies taken in field conditions [18,59,60,61] have not developed such chambers that could separately quantify the N_2_O emitted from the rice plant and the rice soil. In this regard, static chambers that could be used for crops like the rice plant should be developed and the N_2_O fluxes from the rice plant and the rice soil (microbial) should be measured separately. This limitation has made it difficult to evaluate the contribution of the rice plants to the total contribution from the paddy and the N_2_O budget from rice plants remains unsolved. Although it might be a challenge to prove that all N_2_O emitted by the plants in field conditions is from either soil microorganisms, plants, or both, injecting δ ^15^N-N_2_O^bulk^ into the soil profile and subsequently measuring the N_2_O fluxes in the soil and the rice plants might help to distinguish the source. Additionally, to understand the role of the rice plants in the paddy, we suggest measuring the isotopocule fingerprint of N_2_O (δ ^15^N, δ ^15^N^sp^ and δ ^18^O) emitted from rice plants and rice soil, as isotope analysis methods are a more powerful tool than the flux measurement methods to distinguish the source of the N_2_O [63].

At the plant’s cellular level, the NO_3_-NO_2_-NO-N_2_O pathway, as represented in Figure 1 operates during limited oxygen in cells and in the presence of NO_3_, NO_2_ [26,29,30,37,48]. Similarly, in the rice soil the microbial pathway of denitrification (NO_3_-NO_2_-NO-N_2_O-N_2_) occurred during limited oxygen conditions and in the presence of NO_3_ and NO_2_ [10,64]. So, both in the rice plant and the rice soil, the pathway of N_2_O formation occurred during the limited oxygen conditions, making the process appear only to occur in the known site, i.e., the soil. As the N_2_O flux measurement was done with the help of static chambers that captured the N_2_O fluxes in the soil and the rice plant at bulk [18,59], this masked the role of the rice plant. However, as described in Section 2, the NO_3_-NO_2_-NO-N_2_O pathway existed in the plant cells during hypoxia and anoxia and this pathway in the rice plant cells was overlooked.

## 4. Role of NO_3_, NO_2_ and NO during Hypoxia and Anoxia Tolerance

Rice farming needs frequent irrigation, resulting in hypoxic and anoxic conditions in the root zone. Under such conditions, oxygen concentration in the cells is too low to support aerobic respiration and may cause energy deficit and, ultimately, cell death [65]. Various studies have shown that NO_3_ fertilization improves tolerance to low oxygen conditions in plants [31,66]. During hypoxia, and in the absence of NO_3_, the plants’ growth is significantly disturbed [31]. So, for crop species like the rice plant, which is frequently subjected to hypoxia and anoxia, NO_3_ might play important role in improving tolerance to low oxygen.

Oxygen deprivation affects the plant mitochondria [67,68]. Nitrate and nitrite are shown to have a protective effect on mitochondria under oxygen deprivation [68]. Hypoxia strongly decreases the NO_2_ reduction to NH_4_ and the NH_4_ assimilation to amino acids [35]. As NO_2_ assimilated to NH_4_ is reduced during hypoxia [35], it is accumulated in the cytosol [30,65] or released to the external medium [66]. The accumulated NO_2_ can serve as an alternative electron acceptor at mitochondrial electron transport chain (ETC), with NO as a significant product of the reaction [52]. Moreover, the pathway of NO formation through NO_3_-NO_2_ in the mitochondria contributes to the ATP synthesis [33,65,69]. The NO_3_-NO_2_-derived ATP production in mitochondria can make an important contribution to hypoxia survival [70]. Although NO is a signaling molecule [27,28,29,30], an excess of NO in the mitochondria can be toxic to the cell and may result in the death of the cell [71]. Although N_2_O is a potent greenhouse gas, the conversion of NO to N_2_O by cytochrome c oxidase [26,48] might play an important role in maintaining the integrity of mitochondria during limited oxygen conditions, as an excess of NO can be toxic to the cell [71]. So, the high emissions of N_2_O from rice plants during limited oxygen conditions, as reported by Yan et al., (2000) [13] might be an efficient mechanism to reduce the toxicity of NO and to maintain the integrity of the mitochondria under hypoxia and anoxia, therefore, this should be a matter of further research.

## 5. Conclusions and Future Perspectives

In conclusion, NO_3_ is reduced to NO_2_ in the cytosol, and NO_2_ reduction to NO, along with NO reduction to N2O, occurs in the mitochondria of rice plants. The pathway of N_2_O formation is formed under hypoxic or anoxic conditions, and the water management practice may often cause hypoxia and anoxia in the rice root, making the rice plant a potent source of N_2_O. Therefore, we suggest that, while studying N_2_O emission from paddies, the potential pathway of N_2_O formation in rice plants should be explored. To date, the rice plant is hypothesized to be a medium to transport the N_2_O produced in the soil by microorganisms. However, recent studies suggest that plants can produce N_2_O, which may be during the metabolism of NO to N_2_O in plant mitochondria [30], as shown in this study. The significant amount of N_2_O emitted from rice plants suggests the possibility of the existence of the proposed pathway of N_2_O formation in rice plants. Furthermore, the NO_3_-NO_2_-derived NO formation pathway in the hypoxic and anoxic rice plant’s mitochondria helps the ATP synthesis and develop a tolerance to limited oxygen conditions. The rice plant, being an anoxia-tolerant plant, in which NO_2_ dependent NO production in mitochondria was sustained for almost twice as long as barley [67], suggests that these plants might be a more potent producer of N_2_O. However, to date, N_2_O emitted from the paddy is regarded as being produced by various microbes [10,21] and the N_2_O formation pathway in plants is neglected. Future studies are certainly required to elaborate on the proposed pathway of N_2_O formation in the rice plant. Furthermore, the transport of soil-produced N_2_O by plants, as hypothesized by many experiments [12,13], is not supported because there are no emissions of N_2_O by plants when supplied with NH_4_ and during a high rate of N_2_O production in the rhizosphere [15]. So, it would be misleading to understand the N_2_O emitted by the rice plant to be soil microorganism-produced. Understanding the role of the rice plant is necessary for further strategies to be taken to mitigate N_2_O emissions from the paddy. Quantifying the dual isotopocule fingerprint of N_2_O, along with the molecular-based studies, would provide clear insights into the proposed mechanism. Therefore, the extraction of rice mitochondria and the measuring of NO and N_2_O emissions under different conditions of O_2_ and NO_3_ would provide insights into the N_2_O pathway. Besides, the reduced form of cytochrome c oxidase and quinone are found to catalyze NO reduction to N_2_O and, therefore, their roles in rice plants should be investigated in detail. Moreover, to differentiate the soil sources from the plant source and quantify the portion of soil and plant contributions to total emissions will be a big challenge with the current approach to the methodology used. The development of static chambers that could capture the fluxes of N_2_O from the rice plant and separate the soil emissions would help to evaluate the rice plant’s role in the total emissions.
